# Comprehensive Performance Quasi-Non-Volatile Memory Compatible with Large-Scale Preparation by Chemical Vapor Deposition

**DOI:** 10.3390/nano10081471

**Published:** 2020-07-27

**Authors:** Kun Yang, Hongxia Liu, Shulong Wang, Wenlong Yu, Tao Han

**Affiliations:** Key Laboratory for Wide-Band Gap Semiconductor Materials and Devices of Education, The School of Microelectronics, Xidian University, Xi’an 710071, China; kuny2019@163.com (K.Y.); 13772406590@163.com (W.Y.); taohan373@gmail.com (T.H.)

**Keywords:** MoS_2_, non-volatile memory, charge trapped, Raman spectrum, PL spectrum, chemical vapor deposition, hysteresis effect

## Abstract

Two-dimensional materials with atomic thickness have become candidates for wearable electronic devices in the future. Graphene and transition metal sulfides have received extensive attention in logic computing and sensing applications due to their lower power dissipation, so that their processes have been relatively mature for large-scale preparation. However, there are a few applications of two-dimensional materials in storage, which is not in line with the development trend of integration of storage and computing. Here, a charge storage quasi-non-volatile memory with a lanthanum incorporation high-k dielectric for next-generation memory devices is proposed. Thanks to the excellent electron capture capability of LaAlO_3_, the MoS_2_ memory exhibits a very comprehensive information storage capability, including robust endurance and ultra-fast write speed of 1 ms approximately. It is worth mentioning that it exhibits a long-term stable charge storage capacity (refresh time is about 1000 s), which is 10^5^ times that of the dynamic random access memory (refresh time is on a milliseconds timescale) so that the unnecessary power dissipation greatly reduces caused by frequent refresh. In addition, its simple manufacturing process makes it compatible with various current two-dimensional electronic devices, which will greatly promote the integration of two-dimensional electronic computing.

## 1. Introduction

As the size of devices continues to decrease, the difficulty of traditional silicon-based processes continues to increase and is close to its physical limit. There is an urgent need to explore a new channel material to replace silicon, so that Moore’s Law can be continued [1]. At present, the small-size silicon-based transistor uses the Fin-FET structure, which can improve the control ability of the gate to the channel, and greatly improve the performance of the device [2]. Based on this idea, a wide range of researchers have began to seek a suitable two-dimensional semiconductor to effectively improve the integration of the chip [3,4]. Since the discovery of graphene, a large number of layered crystals have been stripped in order to make up for the shortcomings of graphene band gap, therefore the MoS_2_ is a suitable candidate. The energy band structure of MoS_2_ depends on the number of layers and the single-layer MoS_2_ has a direct band gap, which undoubtedly provides huge opportunities for electrical and optoelectronics [5]. In recent years, a lot of research has been carried out surrounding MoS_2_, and various new electronic devices based on MoS_2_ have emerged, including gas sensors, resistive memory, photodetectors, etc. [6,7,8,9,10,11,12]. A lot of research is currently focused on how to make MoS_2_ transistors provide high-performance logic computing power and try to improve the performance of transistors through various process methods, which also brings a series of achievement simultaneously [13,14,15,16]. However, there are few studies on the application of MoS_2_ in storage, and only limited research on MoS_2_ non-volatile memory has been proposed [17,18,19].

With the rapid development of the information age, the huge amount of data presents new challenges to memory technology, especially for storage speed and data retention ability. For volatile memory, the lower integration and high cost have limited its application in cache, whereas the high power consumption obscures the advantages of DRAM due to poor data retention time [20,21]. Therefore, there is an urgent need to propose a comprehensive memory to fill the time-scale gap between volatile and non-volatile memory. So far, the memory based 2D materials were mostly based on the float gate structure with a slow write speed. Simone Bertolazzi et al. proposed a floating gate transistor using graphene as a trap layer with stable information storage capability, but its writing speed is only 100 ms, far lower than the current commercial flush write speed (about 100 µs). Chunsen Liu et al. proposed a kind of semi-floating gate memory with ultra-fast write speed by an embedded p-n junction, but the leakage current of p-n junction limits the date retention time to only 14 s [22,23]. Additionally, the complicated preparation process is based on mechanical peeling making it difficult to prepare on a large scale, which cannot be accepted for commercialization.

In this work, a novel memory device based on the monolayer MoS_2_ was proposed utilizing a lanthanum incorporation high-k dielectric to provide capture traps [24,25]. The memory cells exhibit an impressive performance with large memory windows of more than 3 v (operating voltage from −6 to 6 v and back to −6 v) and high program/erase radio of approximately 10^3^ applied 1 ms voltage pulse. During repeated program/erase operations, no performance degradation is observed and it has great data retention capabilities, indicating that the memory cell is very robust. The proposed device promotes the integration of two-dimensional integrated circuits development trend.

## 2. Experiment Method

### 2.1. The Growth of MoS_2_ by APCVD and Materials Characterization

**Figure 1:**
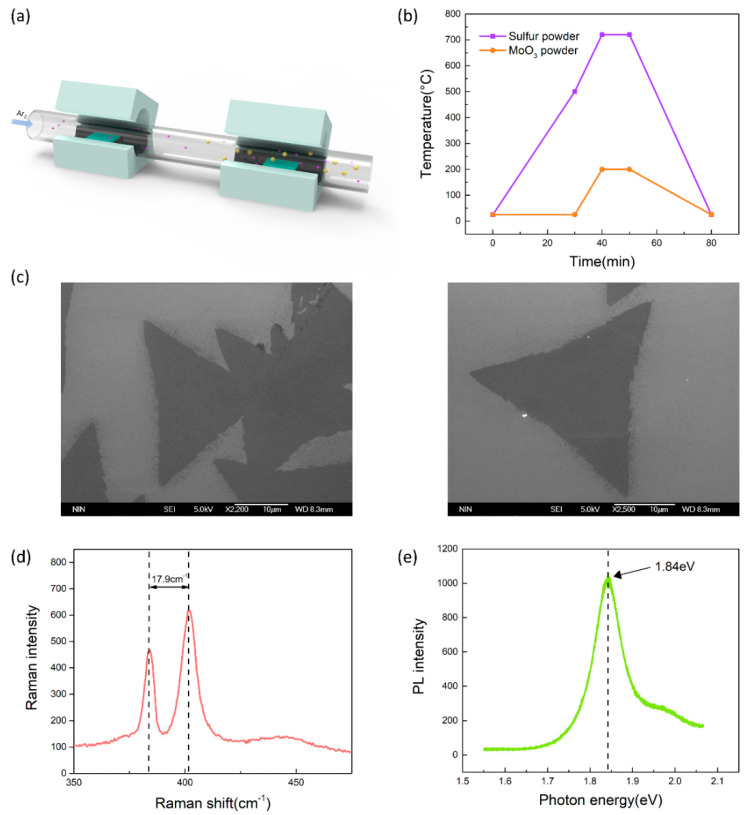
(**a**) The schematic diagram of MoS_2_ synthesis by atmospheric pressure chemical vapor deposition (APCVD). (**b**) Detailed temperature control of the growth process. (**c**) A highly uniform monolayer MoS_2_ in the scanning electron microscope (SEM). (**d**) The Raman spectrum of the monolayer MoS_2_ of two peak positions is 17.9 cm^−1^. (**e**) The PL spectrum of the monolayer MoS_2_ with a 1.84 eV bandgap.

The growth of MoS_2_ uses the traditional atmospheric pressure chemical vapor deposition (APCVD) method by a dual-temperature zone tube furnace, as shown in Figure 1a. The sulfur powder, molybdenum trioxide (MoO_3_) is used as the sulfur source and molybdenum source, respectively. Two quartz boats containing 0.1 g sulfur powder and 2 mg MoO_3_ were put into the tube furnace, where the quartz boat with sulfur powder was located in the low temperature area, and the other quartz boat carrying MoO_3_ and the cleaned substrate was located in the high temperature area. During the growth process, the temperature in the low temperature zone and the high temperature zone are set to 200 and 720 °C, respectively, to ensure that the growth source sublimates. The detailed temperature control of the growth process that can be seen in Figure 1b,c shows the SEM image of the triangle monolayer MoS_2_ by APCVD. The Raman spectroscopy is shown in Figure 1d, exhibiting that the wavenumber difference of two peak positions is 17.9 cm^−1^. Figure 1e shows that the PL intensity at a different energy, from which the peak intensity is located at 1.84 eV, is the typical bandgap of the monolayer MoS_2_ [26].

### 2.2. Device Fabrication and Electronic Characterization

The silicon substrate covered with LaAlO_3_ is used as a new substrate for device fabrication, where La(^i-^PrCp)^3^, TMA as the Al precursor and La precursor, respectively while O_3_ was used as the oxidant. Prior to the deposition of LaAlO_3_ dielectric, native SiO_2_ on the substrate was removed and immersed in a diluted HF solution (HF:H_2_O = 1:50) for 50 s and the HF solution was rinsed out with de-ionized water. Post-deposition rapid thermal annealing was carried under Ar_2_ atmosphere at 800 °C for 60 s. Then, the thickness of LaAlO_3_ was approximately 48 nm measured by the spectroscopic ellipsometry (SE) system. The characteristic of the LaAlO_3_ film was displayed in Figures S1–S4 (in Supplementary Materials). After the high-k dielectric deposition and material synthesis, the source-drain electrode was patterned by electron-beam lithography, followed by Ti (10 nm)/Au(50 nm) deposited by electron-beam evaporation. The electronic characteristic of memory cells was measured by the Agilent B1500A analyzer (Santa Clara, CA, USA) at room temperature.

## 3. Results and Discussion

**Figure 2:**
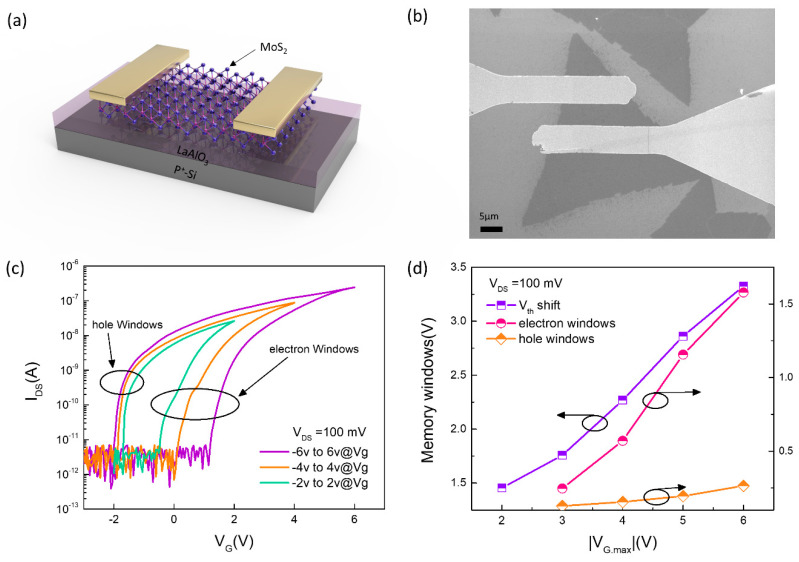
(**a**) The schematic diagram of memory cell with the MoS_2_ channel and LaAlO_3_ charge trapped layer. (**b**) The memory cell in the scanning electron microscope (SEM). (**c**) The double direction transfer characteristic at different sweep voltages. (**d**) The memory windows as a function of the voltage |V_G.MAX_|. The initial threshold corresponding to the threshold voltage sweeping the gate voltage ±2.

**Figure 3:**
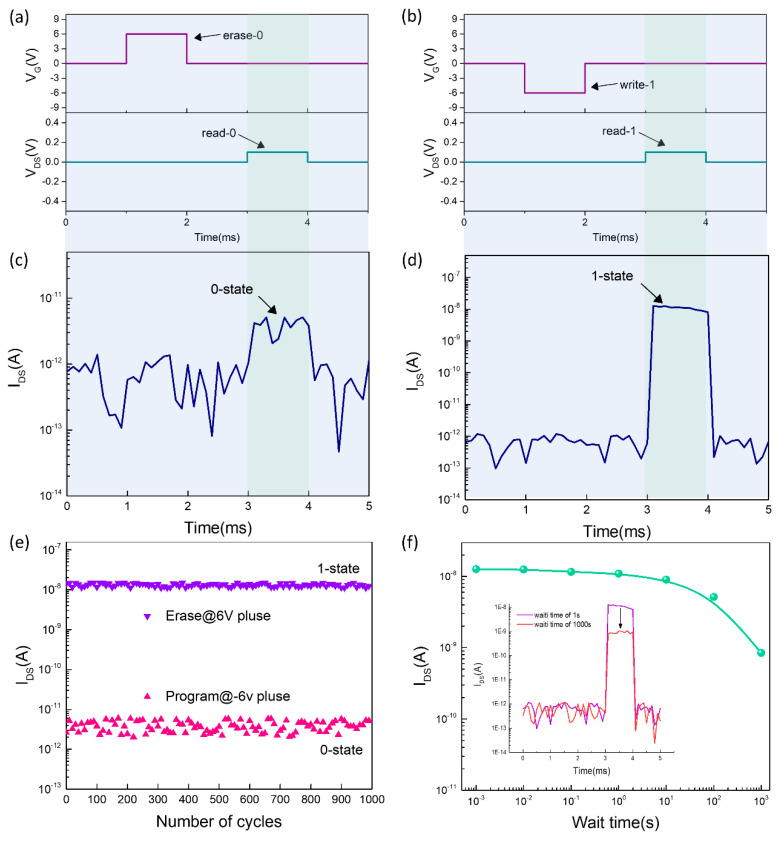
Demonstration of the write/erase operation. (**a**,**c**) The erase operation with +6 v pulse signal and read 0-state, respectively. (**b**,**d**) The program operation with −6 v pulse signal and read 1-state, respectively. (**e**) The endurance testing of memory cell in 1000 cycles, and no performance reduction is observed. (**f**) The exponentially decay characteristic with wait time increasing. The refresh time of 100 s is obtained from the decay curve. The inset of (**f**) shows the output signal degradation when the wait times increase from 1 to 100 s.

Figure 2a shows the diagram of the MoS_2_/LaAlO_3_/P^+^-Si memory cells. In order to analyze the electric characteristic of the device, a double voltage sweep measure was performed. Figure 2b shows the SEM image of the memory cell. The information storage ability of the memory device can be obtained from transfer characteristics at different voltage peaks. As shown in Figure 2c, the on-off ratio up to 1 × 10^4^ can be obtained when the gate voltage was swept from −6 to 6 v. For a better display effect, the *X*-axis coordinates start from −3 v. Even if the no gate voltage is applied, the ratio of forward current and negative current exceeds 1 × 10^3^, enabling the performance of ‘reading-operation’ only applied by the drain voltage, which is essential for reducing power consumption in the integrated circuit. It can be seen from Figure 2c that the threshold voltage drifted to some extent and the threshold voltage drifted more severely with the increase of sweep voltage peaks. When the gate voltage was swept from negative to positive, a large negative voltage was applied to the gate electrode, causing the separation of an electron-hole pair. Due to the smaller effective mass and higher mobility, the electrons transfer to the channel by tunneling under the influence of a strong electric field. On the contrary, a positive high-field makes the electrons tunnel to gate-dielectric. Therefore, with different peak voltages applied, the device threshold voltage will drift at different degrees, determined by the number of electrons tunneling from channel to oxide [27,28,29]. Another typical characteristic of the threshold voltage drift is that the threshold voltage drift is very small when the voltage was swept from negative to positive (hole windows shown in Figure 2c), but when the voltage was swept from positive to negative, the threshold voltage drift is large (electron windows shown in Figure 2c). This is because the tunneling process is mainly led by the electrons, and the holes are not easy to tunnel due to their large effective mass. The relationship between memory windows and sweep voltage is illustrated in Figure 2d, from which we can find obviously that the threshold voltage drifts slightly caused by hole tunneling. The memory window is determined dominantly by electron tunneling, increasing linearly with the rising of the gate voltage. When the gate voltage was swept from −6 to 6 v and back to −6 v, a large memory windows up to 3.3 v is obtained. The large V_th_ shift windows is indispensable for a robust memory device. So as to comprehensively evaluate the storage performance memory cells, we conducted a dynamic storage test. According to the transfer characteristic of the device, by sweeping the voltage from −6 to 6 v and back to −6 v, not only a switch ratio of approximate 1000 can be obtained, but a large memory windows exceeding 3 v ensures the stability of the programming operation. Hence, in the dynamic storage performance test, we chose +6 v, −6 v as the program voltage and erase voltage, respectively. Since a superior switching ratio has been obtained, which allows us still to fix V_D_ = 100 mV as the read voltage. Figure 3a shows the input signal for the erase operation in one cycle, where a rectangular pulse with a peak voltage of 6 v and a pulse width of 1 ms is used for the erase operation. After that millisecond, a rectangular pulse with a peak voltage of 0.1 v and a pulse width of 1 ms is applied to the drain for the read operation. Figure 3c shows the output signal when the device is in the 0-state, from which an output current below 1 × 10^−11^ is obtained for 3–4 ms, but at other times, the current noise signal is detected because of the no read voltage applied. Similarly, a pulse with a peak voltage of −6 v and a pulse width of 1 ms is used for the storage operation, as shown in Figure 3b. When performing the read operation, an output signal of up to 1 × 10^−8^ is obtained for information storage, as shown in Figure 3d. At the same time, we can find that the intensity of the output signal always maintains a good consistency during applying the read voltage with a pulse width of 1 ms, which is vital for information storage. Otherwise, if the output signal is unstable during a read operation, it may cause data loss, especially for the 1-state.

Endurance testing is essential for a high-performance non-volatile memory, which is closely related to the number of programming. Since the programming is mainly dominated by electron tunneling, it is inevitable that the gate oxide layer will be damaged during the process of electron tunneling. For this consideration, we conducted a MoS_2_ memory cell endurance test. In order to speed up the progress of the test during the test, we use the B1510A module to generate a voltage pulse with a width of 1 ms for program and erase and the waiting time is still maintained for 1 ms to guarantee the data retention capability of the memory cell. The reading operation is completed by the I-V-list measurement mode, and only one sampling is performed for the given reading voltage after the waiting time is completed. Figure 3e shows the trend of 1-state current and 0-state current with the number of programming times in a thousand cycles, from which the 1-state current is stability and not any performance reduction is observed. Although the 0-state current produces small fluctuations, it is always lower than 1 × 10^−11^, which guarantees a clear 0–1 state.

Data retention capability is an important indicator for evaluating the performance of non-volatile memory. The capability of data retention is directly related to the frequency at which the memory needs to be powered on and written, which will directly determine the power consumption of the non-volatile memory. The program process is achieved by trapping electrons in the gate oxide layer to change the device turn-on voltage. In practical applications, devices work in various environments so that electrons may obtain energy from external stressing, such as thermal energy, causing random tunneling from gate oxygen to the channel when the electron energy is large enough to cross the barrier height. Therefore, as the final step of device performance characterization, we have studied the data retention capability of the memory, which is directly reflected by changing the waiting time. The traditional method of evaluating memory data retention is from the shift of threshold voltage of the device, determined by the transfer characteristics at different intervals. One disadvantage of this method is that it may cause the device written when sweeping the transfer characteristic curve, thereby causing inaccuracy of the data. For the charge retention test, we adopted a simple and accurate method, which ensures the accuracy of the test results. After performing a program operation (+6 v pulse during 1 ms), the 1-state current of the memory is measured only by applying a drain voltage of 100 mV at different waiting times. As shown in Figure 3f, the 1-state current decays exponentially with the increasing wait time, fitted well with $I_{DS}=I_{0}+A\times e^{-\frac{t}{\tau }}$ [23]. Nothing but, when the wait time is 100, the 1-state current only shows a slight drop, which can still guarantee the accuracy of the stored information. Compared with the poor data storage capacity of volatile memory (approximately on the millisecond timescale) [29], the refresh time is increased by about 10^5^ times, which will greatly reduce the power consumption of the memory.

**Figure 4:**
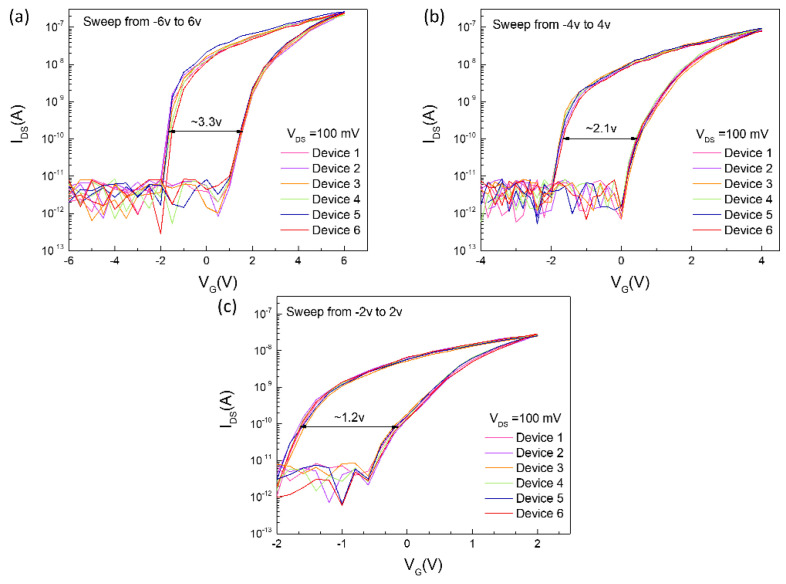
Device-to-device repeatability test from six different regions. (**a**) Sweeping in ± 6 v. (**b**) Sweeping in ± 4 v. (**c**) Sweeping in ± 6 v.

It is well known that the interface state charge may cause the hysteresis effect, which induces the threshold voltage variation. The hysteresis effect induced by the interface trap charge is always uncontrollable. In order to better illustrate that the device performance obtained is not accidental, we conducted a device repeatability test, as shown in Figure 4. The six devices we selected are distributed in different areas of the same sample. Memory windows sweeping is performed at different sweep voltages for all devices. From Figure 4, these six devices show good repeatability, although there is a little fluctuation in the transfer characteristic, the influence of these fluctuations on the memory cell is negligible. Finally, we compared the device performance with other literatures, exhibited in Table 1. It can be found that the device we proposed not only can obtain a comparable device performance with the other memory, but also greatly simplifies the manufacturing process, which provides the possibility for large-scale manufacturing.

## 4. Mechanism Analysis

**Figure 5:**
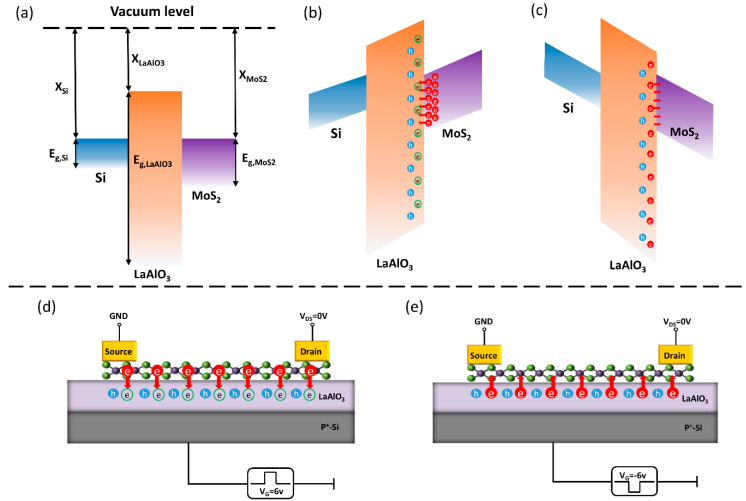
(**a**) The energy band diagram without the applied voltage (X_Si_ ≈ 4.13 eV, E_g,Si_ ≈ 4.13 eV, X_LaAlO3_ ≈ 2.39 eV, E_g,LaAlO3_ ≈ 6.5 eV, X_MoS2_ ≈ 4.2 eV, E_g,MoS2_ ≈ 1.8 eV). (**b**,**d**) The transfer of electrons in the program operation. (**c**,**e**) The transfer of electrons in the erase operation.

The current main charge storage devices are based on floating gate structures [22,23,30,31], as shown in Figure S5. During the programming process, the electrons tunnel pass through the barrier layer and are trapped by the floating gate, whereas electrons are de-trapped during the erase operation. The trapping layer plays a leading role for charge storage. Due to the better compactness and higher crystallinity of binary oxides, there are only a few trap states in binary oxides. Therefore, the contribution of the charge restrained by the trap state to the operating window is insignificant. However, for the memory device we proposed, there is not the capture layer. Lanthanum incorporation in Al_2_O_3_ broke the original lattice structure of Al_2_O_3_, causing a mass of electron traps to exist in LaAlO_3_, as shown in Figure 5b. The green hollow circle represents the electron trap in oxide. During the programming process, the electron trap is filled with electrons, and offset with the fixed charge of the oxide layer (blue solid circles). This electron transfer process causes the threshold voltage to drift.

The hysteresis effect in the MoS_2_ transistor based on SiO_2_ may be dominant by the interface trap charge, because it is seriously affected by environmental factors [31,32]. That will not have good device-to-device reproducibility, whereas the devices we proposed exhibit good repeatability, as shown in Figure 4. Moreover, in some literatures, Al_2_O_3_ encapsulation is used to reduce the hysteresis effect [33]. Although the hysteresis effect in the MoS_2_ transistor based on SiO_2_ has a false effect that is similar to a memory device, there are many shortcomings in SiO_2_. As shown in Figure S6, despite having a large memory windows, the program/erase ratio is still less than 1 × 10^2^ due to the small dielectric constant of SiO_2_. Not only that, the program/erase voltage has reached ±40, which is disadvantageous for the endurance of memories. However, our proposed memory cell only needs a ±6 operation voltage to achieve a program/erase ratio of 1 × 10^3^, which shows great advantages in storage applications.

## 5. Conclusions

In this work, we have manufactured a charge trapped non-volatile memory based on MoS_2_ transistors with extremely strong information storage capabilities. This memory cell exhibits a large memory windows of 3.3 v (sweeping from −6 to 6 v and back to −6 v), ultra-fast writing speed (up to 1 ms), stable program/erase ratio of 10^3^, and longer refresh time (approximately 100 s) that avoids unnecessary power dissipation due to frequent refresh operations. At the same time, the simple back-gate process makes it well compatible with existing memory fabrication processes. All these impressive performances indicate that the charge trapping non-volatile memory based on MoS_2_ transistors will promote the development of a two-dimensional memory technology.

## Figures and Tables

**Table 1:** Comparison of the performance of memory with other literatures.

Ref.	Materials	Program/Erase Ratio	Program/Erase Voltage	Write Speed	Date Retention Time	Process Complexity	Large-Scale Preparation
Our result	MoS_2_/LaAlO_3_	~1 × 10^3^	±6 v	1 ms	1000 s	I	√
[22]	HfO_2_/Graphene/HfO_2_/MoS_2_	~1 × 10^3^	±18 v	100 ms	10 year	II	×
[23]	HfS_2_/MoS_2_/BN/WSe_2_	~1 × 10^2^	±5 v	15 ns	10 s	IV	×
[27]	Al_2_O_3_/HfO_2_/Al_2_O_3_/MoS_2_	<1 × 10^3^	±26 v	200 ms	-	II	×
[29]	MoS_2_/BN/WSe_2_	~1 × 10^2^	±5 v	40 ns	14 s	III	×
[30]	HfO_2_/Metal/HfO_2_/MoS_2_	~1 × 10^5^	±15 v	100 ms	10 year	II	×

Process complexity progressively increases from I to IV.

## Supplementary Materials

The following are available online at https://www.mdpi.com/2079-4991/10/8/1471/s1, Figure S1: LaAlO3 film interface atomic force microscopy (AFM) topography, Figure S2: XPS spectrum of LaAlO3 film, Figure S3: O1s XPS spectrum of LaAlO3 film, Figure S4: Cross-sectional HRTEM images of sample, Figure S5: Non-volatile Memory Cells Based on MoS2/Graphene Heterostructures, Figure S6: MoS2/SiO2 back gate transistor.
