# Supplementary material for: Comprehensive Performance Quasi-Non-Volatile Memory Compatible with Large-Scale Preparation by Chemical Vapor Deposition
Source: Nanomaterials (Basel). 2020 Jul 27;10(8):1471. doi: 10.3390/nano10081471 (PMC7466503; doi:10.3390/nano10081471)
Supplement: Supplementary file 1 [file nanomaterials-10-01471-s001.pdf]

nanomaterials

Supplementary Materials

Comprehensive Performance Quasi-Non-Volatile Memory Compatible with Large-Scale Preparation by Chemical Vapor Deposition

Kun Yang, Hongxia Liu *, Shulong Wang *, Wenlong Yu and Tao Han

Key Laboratory for Wide-Band Gap Semiconductor Materials and Devices of Education, The School of Microelectronics, Xidian University, Xi'an 710071, China; kuny2019@163.com (K.Y.); 13772406590@163.com (W.Y.); taohan373@gmail.com or 15639119745@163.com (T.H.)

* Correspondence: hxliu@mail.xidian.edu.cn (H.L.); slwang@xidian.edu.cn (S.W.); Tel.: +86-130-8756-8718 (H.L.); +86-150-9115-4611 (S.W.)

Figure S1 shows the surface topography of LaAlO3 with a scan size of 1×1μm2, and the 3D topography image is shown in Figure S1b. The root mean square of the film surface roughness is about 1.82 nm. According to the literature [1], proper surface roughness is conducive to improving device performance, which is related to the strain of MoS2. At the same time, literature [2] pointed out that rough surface will produce greater threshold voltage drift than smooth surface. This may also be the part reason why we get the ideal memory device. Figure S2 shows the XPS spectrum of LaAlO3 film, the binding energy positions of La3d, O1s, Al2p are 843.17eV, 536.17eV, 78.67eV, respectively. Figure S3 shows the O1s XPS spectrum of LaAlO3 film. The O1s XPS spectrum is fitted with two Gauss-Lorentz peaks. The binding energy positions of La-O-Al and Al-O-Al are 530.27 eV and 531.67 eV, respectively. Figure S4 shows the HRTEM image of the sample of LaAlO3 film, from which an unclear transition layer is found that is caused by Si element diffusion. No nano-sized crystals or long-range ordered crystals are observed in LaAlO3 film, which indicates that the LaAlO3 film may be amorphous.

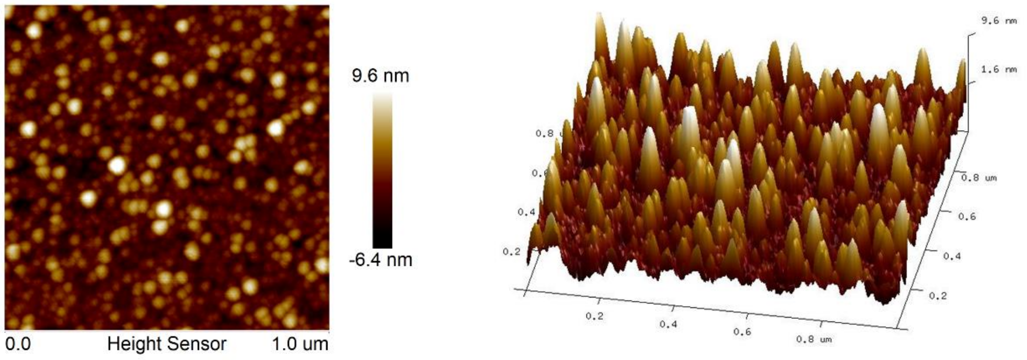

Figure S1. LaAlO3 film interface atomic force microscopy (AFM) topography. (**a**) 2D image. (**b**) 3D image.

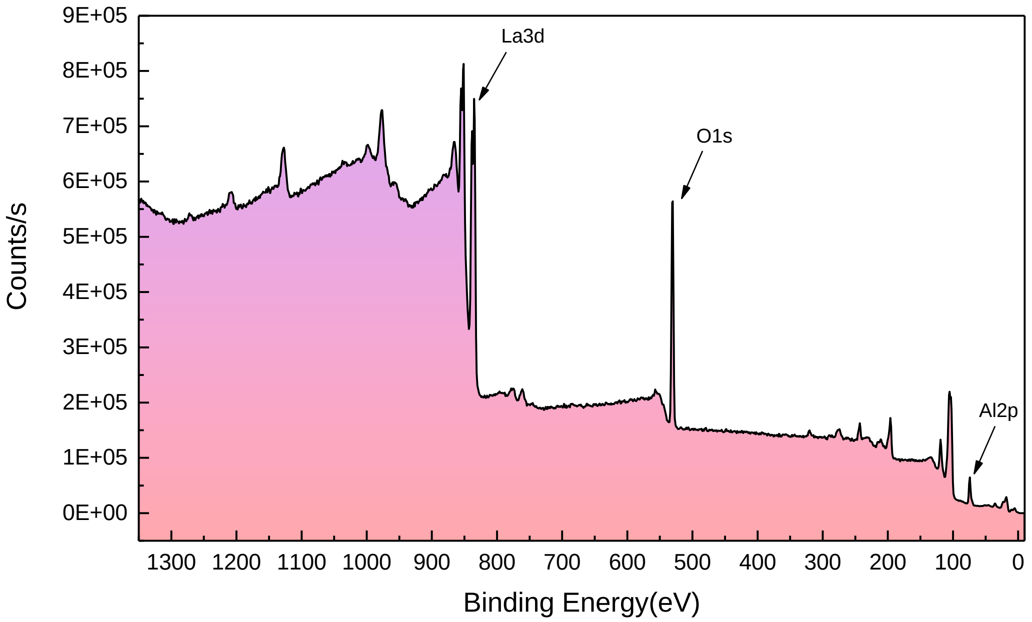

Figure S2. XPS spectrum of LaAlO3 film.

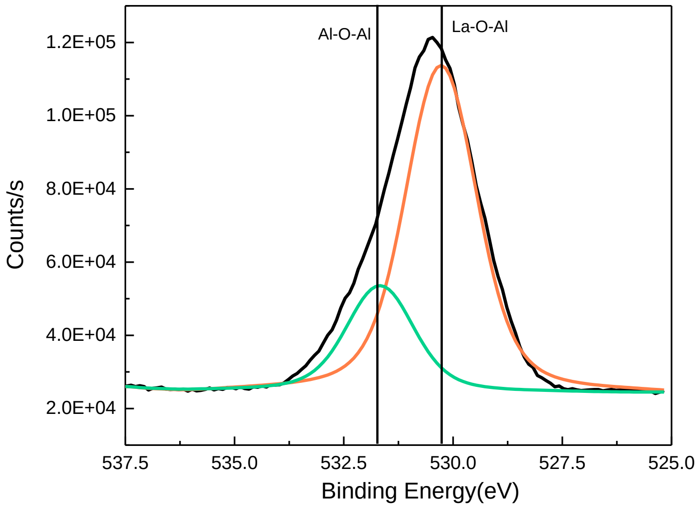

Figure S3. O1s XPS spectrum of LaAlO3 film.

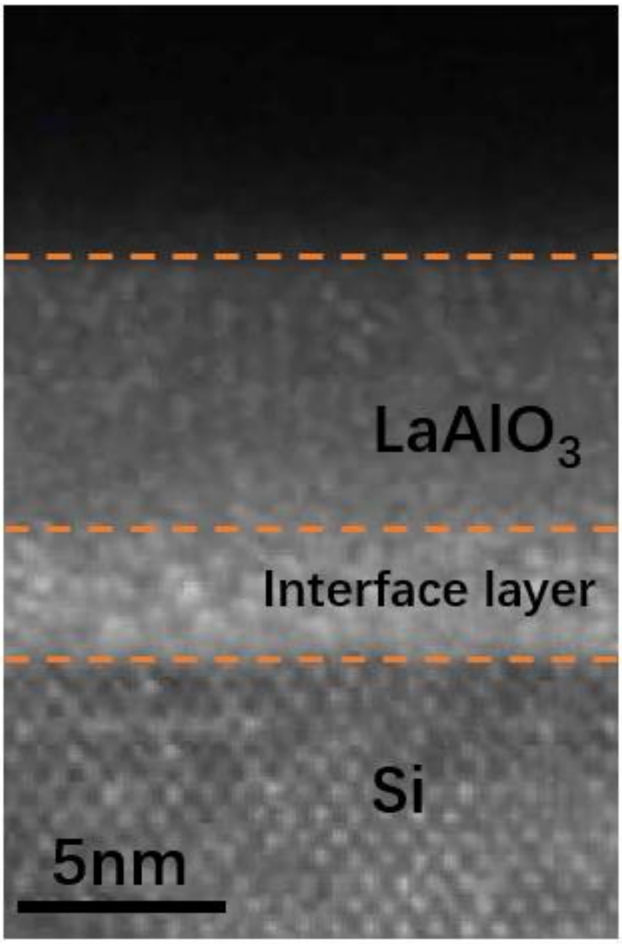

Figure S4. Cross-sectional HRTEM images of sample.

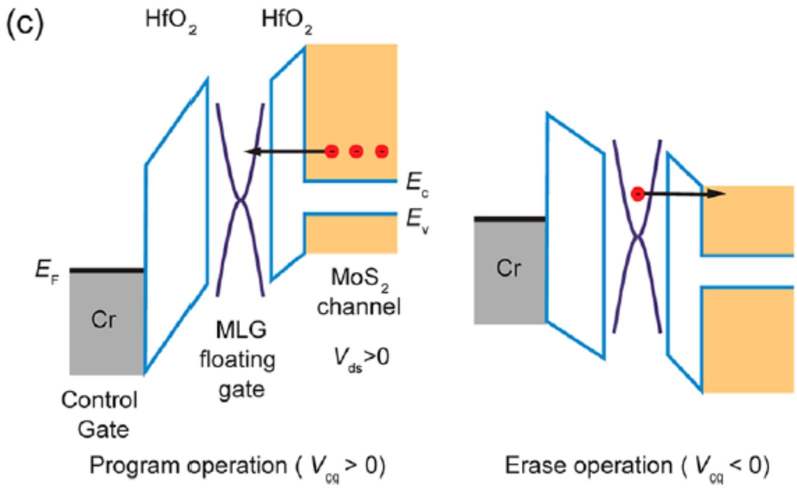

Figure S5. Non-volatile Memory Cells Based on MoS2/Graphene Heterostructures [3].

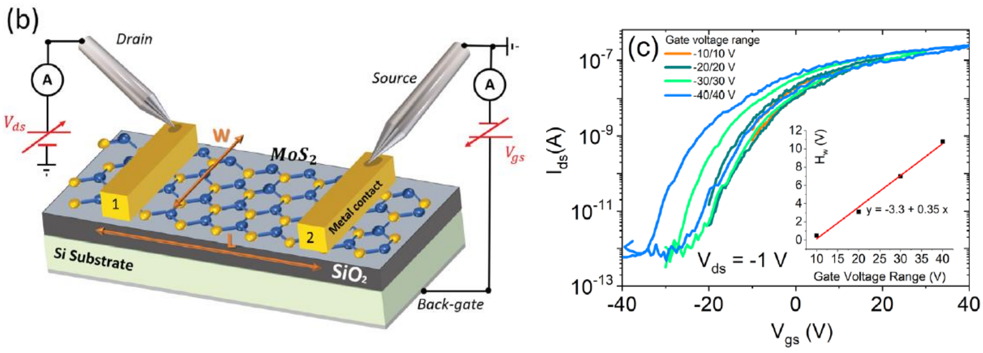

Figure S6. MoS2/SiO2 back gate transistor [4].

References

1. Liu, T.; Liu, S.; Tu, K.H.; Schmidt, H.; Chu, L.; Xiang, D.; Martin, J.; Eda, G.; Ross, C.A.; Garaj, S. Crested two-dimensional transistors. *Nat Nanotechnol* **2019**, *14*, 223–226.
2. Lu, Z.; Serrao, C.; Khan, A.I.; You, L.; Wong, J.C.; Ye, Y.; Zhu, H.; Zhang, X.; Salahuddin, S. Nonvolatile MoS2 field effect transistors directly gated by single crystalline epitaxial ferroelectric. *Appl. Phys. Lett.* **2017**, *111*, 023104.
3. Bertolazzi, S.; Krasnozhon, D.; Kis, A. Nonvolatile Memory Cells Based on MoS2/Graphene Heterostructures. ACS Nano **2013**, *7*, 3246–3252.
4. Di Bartolomeo, A.; Genovese, L.; Giubileo, F.; Iemmo, L.; Luongo, G.; Foller, T.; Schleberger, M. Hysteresis in the transfer characteristics of MoS2 transistors. 2D Materials **2017**, *5*, 015014.

© 2020 by the authors. Licensee MDPI, Basel, Switzerland. This article is an open access article distributed under the terms and conditions of the Creative Commons Attribution (CC BY) license (http://creativecommons.org/licenses/by/4.0/).
